# Dual effects of JAK vs TNF inhibitors on osteoporosis, fractures and mortality in rheumatoid arthritis: a real-world cohort study

**DOI:** 10.3389/fimmu.2026.1745048

**Published:** 2026-03-18

**Authors:** Chunyan Huang, Daorong Hong, Yao-Min Hung, Shiow-Ing Wang, James Cheng-Chung Wei, Xiaoqing Chen

**Affiliations:** 1 Department of General Practice, The Second Affiliated Hospital of Fujian Medical University, Quanzhou, Fujian, China; 2 Department of Ultrasonography, The Second Affiliated Hospital of Fujian Medical University, Quanzhou, Fujian, China; 3 Department of Internal Medicine, Taitung Hospital, Ministry of Health and Welfare, Taitung, Taiwan; 4 Center for Health Data Science, Department of Medical Research, Chung Shan Medical University Hospital, Taichung, Taiwan; 5 Department of Allergy, Immunology & Rheumatology, Chung Shan Medical University Hospital, Taichung, Taiwan Graduate Institute of Integrated Medicine, China Medical University, Taichung, Taiwan

**Keywords:** all-cause mortality, JAK inhibitors (JAKi), osteoporosis, rheumatoid arthritis (RA), tumor necrosis factor alpha inhibitors (TNFi)

## Abstract

**Purpose:**

This real-world study compared the effects of JAK inhibitors (JAKi) versus TNF inhibitors (TNFi) on bone health and survival in rheumatoid arthritis (RA) patients.

**Methods:**

We conducted a retrospective cohort study using the collaborative electronic health records (EHR) database network (2016-2024). After 1:1 propensity score matching, 16, 572 JAKi and 16, 572 TNFi users were included, with follow-up for up to 5 years. Primary outcomes were a composite of “any fracture or osteoporosis, ” individual fracture and osteoporosis events, and all-cause mortality.

**Results:**

In the propensity score–matched cohort, JAKi use was associated with a lower risk of the composite outcome (HR = 0.930) and osteoporosis (HR = 0.906) compared with TNFi. However, a reduction in fracture risk was not clearly observed. JAKi use was also associated with higher all-cause mortality (HR = 1.582). Subgroup estimates suggested potential heterogeneity, but these findings were exploratory.

**Conclusion:**

In this large EHR-based cohort, JAKi initiation was associated with lower rates of osteoporosis-related outcomes but higher all-cause mortality compared with TNFi. Given the observational design, potential residual confounding, and limited follow-up for many patients, these findings should be interpreted as associations and warrant confirmation in other datasets and prospective studies.

**Rationale:**

To compare real-world associations of JAKi vs TNFi with bone outcomes and all-cause mortality in RA.

**Main result:**

In a propensity score–matched cohort, JAKi use was associated with lower osteoporosis-related outcomes but higher all-cause mortality compared with TNFi.

**Significance:**

These observational findings suggest a potential trade-off and highlight the need for individualized risk–benefit discussions and confirmation in other datasets/prospective studies.

## 1 Introduction

Rheumatoid arthritis (RA) is a complex, systemic autoimmune disease characterized by chronic, symmetric, progressive polyarthritis (1, 2). Compared to non-RA individuals, the incidence of osteoporosis in RA patients is as high as 50%, and the risk of fractures is 2 to 3 times greater (3). Chronic systemic inflammation, immune dysregulation and corticosteroid treatment associated with RA (4, 5). Both have complex effects on bone metabolism. Existing studies have shown that disease-modifying anti-rheumatic drugs (DMARDs), through systemic immune suppression, may help reduce bone loss and fracture risk in RA patients to some extent (6).

In recent years, targeted therapies such as Janus kinase inhibitors (JAKi) and tumor necrosis factor alpha inhibitors (TNFi) have made significant progress in the treatment of RA (7). These therapies are also recommended by the ACR and EULAR for moderate to severe patients with inadequate disease control (8, 9). The JAK/STAT pathway plays a key role in the pathogenesis of many inflammatory and autoimmune diseases, including RA. JAKi are thought to block the JAK/STAT signaling pathway, regulating various cytokines such as IL-6 and IL-17, and potentially promote bone formation by activating the Wnt/β-catenin signaling pathway (10). At the same time, JAKi inhibits osteoclastogenesis by suppressing the expression of RANKL in osteoblasts, thus demonstrating a unique bidirectional regulatory effect in the prevention and treatment of osteoporosis (11). As a traditional biologic targeted therapy, TNFi specifically inhibits the TNF-α/NF-κB signaling pathway, reducing osteoclast activity, thus, it effectively inhibits bone resorption (12). However, while both drug classes suppress inflammation, they may also weaken immune defenses, leading to adverse events. JAK inhibitors have been associated with a broader range of adverse effects, including infections, cardiovascular events, and thrombosis, whereas TNF inhibitors are primarily linked to an increased risk of serious infections (13, 14).

Although JAKi and TNFi have shown significant effectiveness in the treatment of RA, direct comparative studies on their effects on osteoporosis, fractures, and all-cause mortality are still scarce, especially in terms of their specific impact on bone metabolism and individualized risk management. This study utilizes a collaborative electronic health records (EHR) database, which covers a large RA patient population, to present the double-edged sword effect of JAKi and TNFi through real-world data analysis. This article fills the gap in direct comparative research on JAKi and TNFi regarding osteoporosis, fractures, all-cause mortality risks, and personalized treatment, while providing detailed risk analysis and population stratification data. This study offers new insights into optimizing personalized RA treatment strategies. Although these findings may provide insights for future research and policy development, it is important to note that, as an observational study, further validation through other datasets and prospective studies is needed.

## 2 Methods

### 2.1 Study design and data source

**Figure 1:**
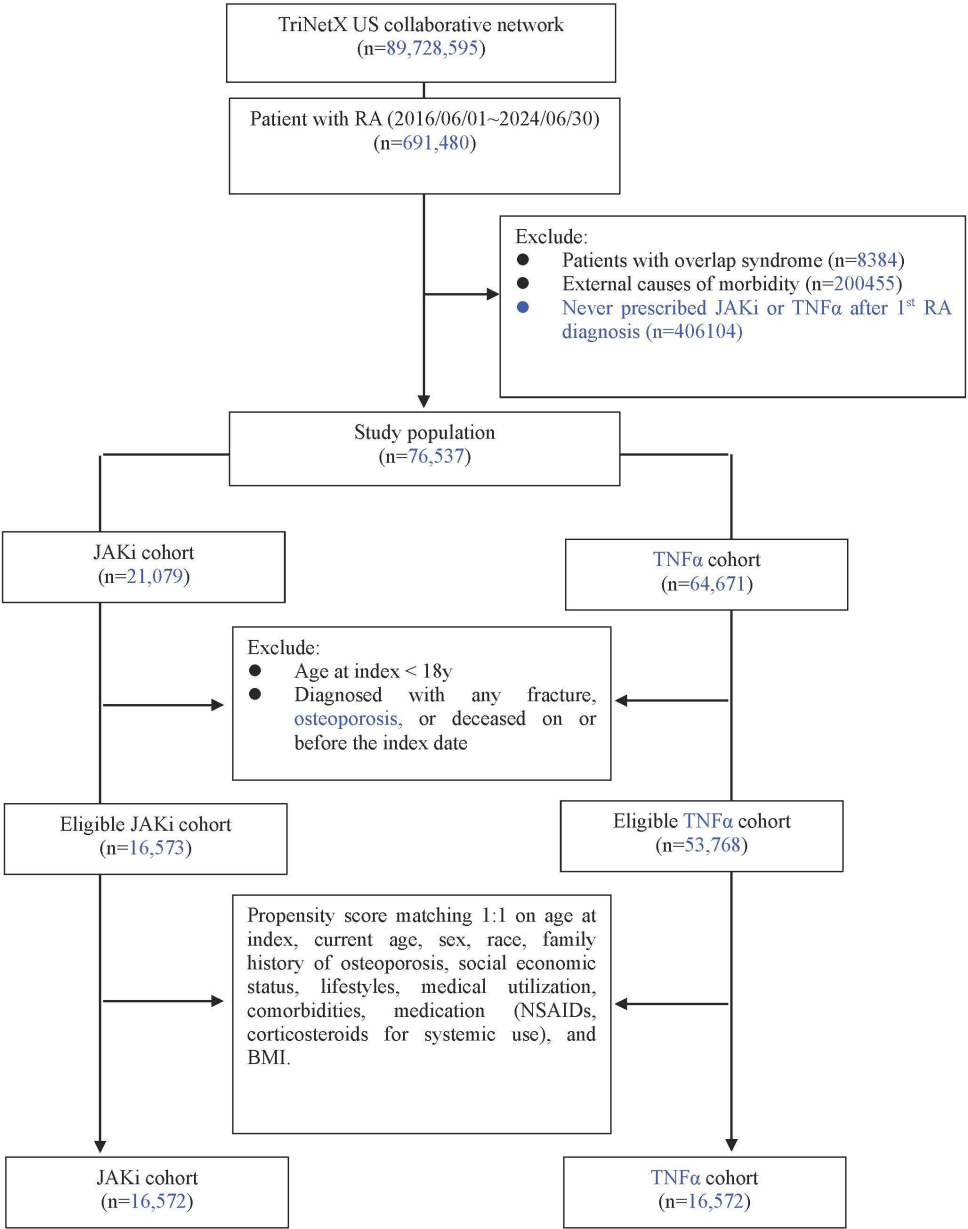
Selection flow and study design.

This study is a retrospective cohort study based on real-world data, sourced from a collaborative electronic health records (EHR) database. We analyzed RA patients (ICD-10 codes M05/M06) with up to 5-year follow-up data encompassing demographic characteristics, medication histories, diagnostic codes, laboratory results, and clinical outcomes to compare osteoporosis, fracture, and mortality risks between Janus kinase inhibitors (JAKi; including tofacitinib, baricitinib, and upadacitinib) and TNF-α inhibitors (TNFi; including adalimumab, etanercept, infliximab, certolizumab pegol, and golimumab). Given the limited available data on individual JAK inhibitors and the need for sufficient statistical power, we pooled all JAK inhibitors together for this analysis. However, we acknowledge that this approach may mask potential drug-specific differences, and future studies with larger sample sizes may benefit from analyzing the effects of individual JAK inhibitors on bone metabolism separately. We applied an active-comparator, new-user design using an as-treated framework within a collaborative EHR database. The index date was defined as the first recorded prescription/medication record of the index class (JAKi or TNFi). We required at least 12 months of observable EHR activity prior to the index date to define baseline covariates and excluded patients with any record of the index class during this baseline window (12-month washout for the index class). Prior exposure to other biologic/targeted synthetic DMARD classes (including the comparator class) prior to the index date was allowed, provided that patients met the new-initiator criterion for the index class during the baseline window. Patients were followed from initiation until the earliest of the outcome of interest, death, end of available follow-up, or switching/discontinuation as captured in the EHR. The intermediate sample sizes shown in **Figure 1** (e.g., 21, 079 JAKi) represent class-specific initiator cohorts prior to baseline exclusions (e.g., age ≥18 years and exclusion of outcomes before or on the index date), whereas the eligible cohorts for matching were 16, 573 (JAKi) and 53, 768 (TNFi). After 1:1 propensity score matching, the final analytic cohort included 16, 572 patients in each group.

### 2.2 Reliability and academic support of the EHR database

Data in the EHR database are de-identified in compliance with HIPAA and GDPR (15). Analyses are executed within the secure collaborative EHR platform using de-identified patient-level EHR data contributed by participating healthcare organizations. However, investigators do not receive or export record-level datasets or individual identifiers; only aggregate outputs (e.g., cohort counts, summary statistics, Kaplan–Meier curves, and model estimates such as HRs with 95% CIs) are accessible. Therefore, informed consent is waived as the dataset is de-identified and investigator access is limited to aggregated results. The collaborative EHR platform has been widely used in retrospective real-world studies (16, 17).

### 2.3 Study participants and matching

The study participants and matching process were based on the EHR platform, using data collected from a total of 89, 728, 595 individuals between June 1, 2016, and June 30, 2024 (**Figure 1**). After excluding individuals with malignant tumors and those who had died prior to the index date, 691, 480 patients diagnosed with RA were included. We identified RA patients using ICD-10 codes M05 (seropositive RA) and M06 (other RA), which have demonstrated 92% positive predictive value in prior validation studies (18). To ensure diagnostic accuracy, we required: 1. ≥2 RA diagnoses from rheumatologists. 2. Concurrent prescription of DMARDs. 3. Exclusion of overlap syndromes (ICD-10 M35) (18).

The accuracy and applicability of ICD-10 coding have been confirmed in multiple real-world data (RWD) studies. Research based on the EHR database has demonstrated that ICD-10 codes have high clinical utility in cohort construction and disease prediction (19). This study adopts strict inclusion and exclusion criteria and integrates EHR real-world data (RWD) to minimize the risk of ICD-10 coding errors, ensuring that the included RA patient cohort is highly representative and reliable.

### 2.4 Propensity score matching

To account for confounding factors, we performed propensity score matching (PSM). The propensity score was estimated using logistic regression based on baseline characteristics, including [list of variables]. We matched individuals in the treatment and control groups based on their propensity scores.

### 2.5 Matching and balance diagnostics

To reduce confounding bias, we performed propensity score matching (PSM) and set a standardized mean difference (SMD) threshold of <0.1 as the acceptable criterion for balance between groups. Variables with an SMD greater than 0.1, such as smoking and depression, were further considered in the subsequent sensitivity analysis to assess their potential impact.

### 2.6 Drug exposure

Drug exposure was defined using an as-treated approach. The index date was the first recorded prescription/medication record of JAKi or TNFi. Patients were followed from initiation until the earliest of the outcome event of interest, documented switching to the comparator advanced-therapy class (TNFi→JAKi or JAKi→TNFi), death, or end of available follow-up. Patients who switched therapies were censored at the crossover date and were not re-assigned to the alternative cohort in the primary analysis. Patients with a history of the outcome of interest prior to the index date were excluded. Medications were identified using EHR medication concepts mapped to RxNorm codes. Based on EHR follow-up data, the median follow-up time was 197 days in both cohorts.

To account for the potential misclassification of exposure due to the different dosing schedules of JAKi (daily oral administration) and TNFi (heterogeneous dosing intervals), we used fixed prescription gaps (≤90 days for general adherence and ≤60 days for “strict adherence”). We acknowledge that these thresholds may introduce differential exposure misclassification between the drug classes. Sensitivity analyses were performed to assess whether alternative exposure definitions based on the varying administration schedules of these drug classes would impact the primary outcomes.

### 2.7 Fracture ascertainment

Fractures were identified based on ICD-10 codes and confirmed through corresponding imaging reports (X-ray/CT/MRI). These reports were assessed by trained radiologists who reviewed the imaging studies and classified fractures according to standard diagnostic criteria. The inclusion of imaging reports ensured the accurate identification of fractures, including those not immediately apparent on clinical examination.

Non-vertebral fractures: Non-vertebral fractures were identified using ICD-10 codes for specific fracture sites, including the hip (S72.0), distal radius (S52.5), and other major non-vertebral fractures. Fractures related to trauma were excluded from the analysis. This approach allowed for a clear distinction between osteoporotic fractures and those resulting from trauma.

### 2.8 Handling of competing risks

In the as-treated analysis, death was treated as a censoring event. Formal competing risk models were not employed, as the current analytic tools within the collaborative EHR platform did not support this approach.

### 2.9 Outcome ascertainment

Primary outcomes were rigorously defined:

a. Osteoporosis:

ICD-10 M80/M81 PLUS.

DXA scan showing T-score ≤-2.5 at lumbar spine/femoral neck.

Or rheumatologist-confirmed diagnosis.

b. Fractures:

ICD-10 codes for vertebral (S22.0, S32.0), hip (S72.0), or non-vertebral fractures PLUS.

Corresponding imaging reports (X-ray/CT/MRI).

Excluded traumatic fractures (ICD-10 Chapter XX).

c. Mortality:

Social Security Death Index linkage.

Hospital discharge status.

### 2.10 Propensity score matching

To minimize confounding bias, propensity score matching (PSM) was used to match patients in the JAKi group and TNFi group on a 1:1 basis. The matching variables included: ① age, sex, race, body mass index (BMI); ② disease duration and severity of RA; ③ socioeconomic status and lifestyle factors (such as smoking and alcohol consumption); ④ comorbidities (such as diabetes, hypertension, and chronic kidney disease); ⑤ baseline medication use (such as nonsteroidal anti-inflammatory drugs (NSAIDs) and corticosteroids). After matching, no significant differences were found between the two groups in baseline characteristics (standardized mean difference (SMD) < 0.1).

### 2.11 Statistical analysis

**Figure 2:**
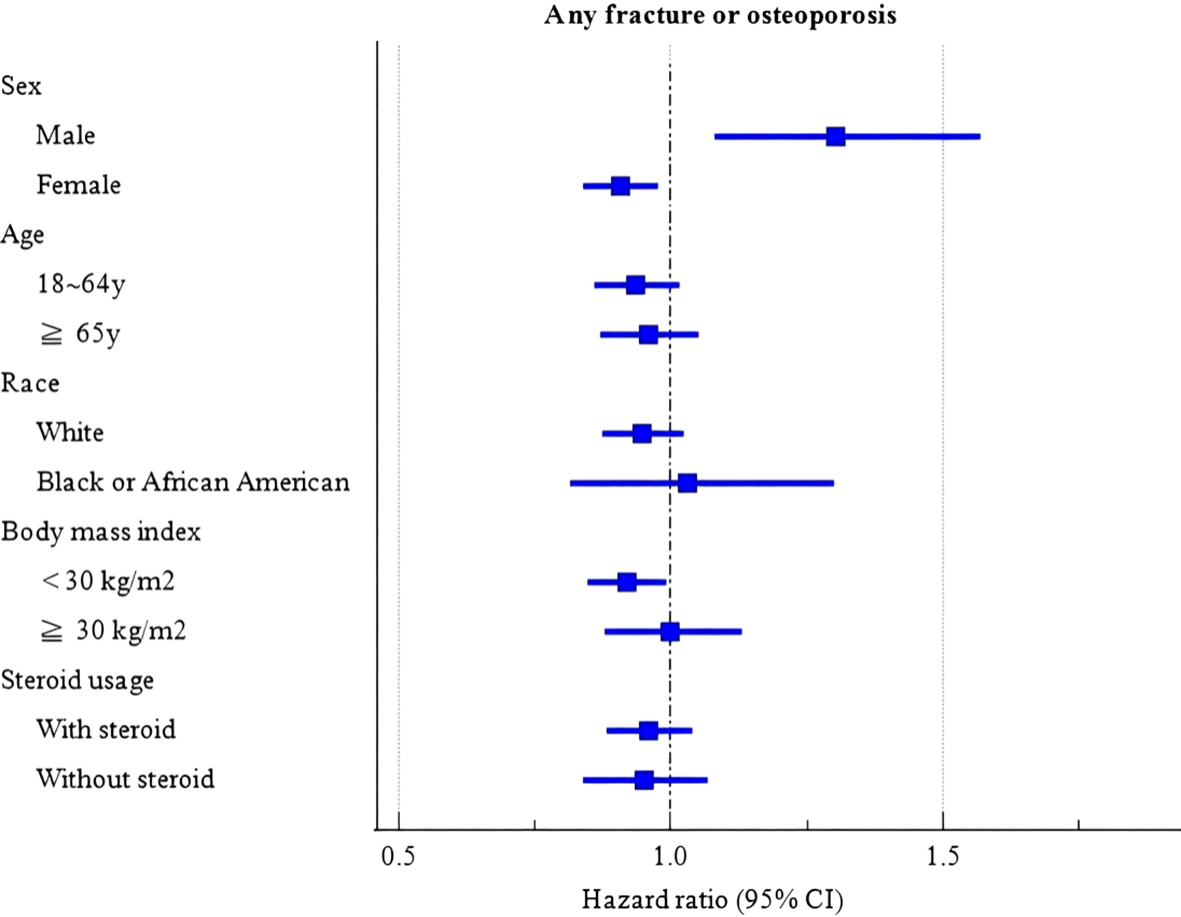
Forest plot of subgroup analyse.

Statistical analyses were performed using EHR’s internal analytics tools. Baseline characteristics between the JAKi and TNFi groups were compared using chi-square tests, and standardized mean differences (SMDs) were calculated; an SMD <0.1 was considered indicative of adequate balance after propensity score matching (PSM). Kaplan–Meier analysis was used to assess the incidence of “any fracture or osteoporosis” in both groups, and log-rank tests were performed to evaluate intergroup differences. Hazard ratios (HRs) with 95% confidence intervals (CIs) were estimated using Cox proportional hazards models implemented within EHR. Subgroup analyses (**Figure 2**) for the composite endpoint of “any fracture or osteoporosis” were conducted by fitting separate Cox models within each prespecified stratum (sex [male vs female], age [18–64 vs ≥65 years], race [White vs Black or African American], BMI [<30 vs ≥30 kg/m²], and steroid use [user vs non-user]) in the matched cohort. We did not perform formal treatment-by-subgroup interaction tests (i.e., interaction terms in a single model); therefore, subgroup findings are presented as exploratory and descriptive. Sensitivity analyses included restricting to patients with ≥2 prescriptions and excluding therapy switchers during follow-up. A two-sided P value <0.05 was considered statistically significant.

## 3 Results

### 3.1 Baseline characteristics of the study population

Prior to propensity score matching, the JAKi were younger, with higher proportions of White individuals, hypertension, dyslipidemia, and metabolic comorbidities compared to TNFi (**Table 1**). After 1:1 matching (n=16, 572 per group), standardized mean differences (SMD) were <0.1 for all covariates except depression (SMD = 0.12) and smoking (SMD = 0.15), indicating adequate balance. (**Table 1**).

**Table 1:** Baseline characteristics of study subjects (before and after PSM matching).

Variables	Before PSM			After PSM_a_		
	JAKi cohort (n=16573)	TNFα cohort (n=53768)	SMD	JAKi cohort (n=16572)	TNFα cohort (n=16572)	SMD
Age at Index, y						
Mean ± SD	54.1 ± 13.6	52.7 ± 14.5	0.099	54.1 ± 13.6	54.0 ± 14.1	0.012
Current Age, y						
Mean ± SD	58.3 ± 13.8	57.6 ± 14.7	0.049	58.3 ± 13.8	58.1 ± 14.3	0.014
Sex, n (%)						
Female	12643 (76.3)	38476 (71.6)	0.108	12642 (76.3)	12732 (76.8)	0.013
Male	3391 (20.5)	13550 (25.2)	0.113	3391 (20.5)	3332 (20.1)	0.009
Unknown Gender	539 (3.3)	1742 (3.2)	0.001	539 (3.3)	508 (3.1)	0.011
Race, n (%)						
White	11641 (70.2)	37504 (69.8)	0.011	11640 (70.2)	11820 (71.3)	0.024
Black or African American	1803 (10.9)	5710 (10.6)	0.008	1803 (10.9)	1760 (10.6)	0.008
Other Race	768 (4.6)	2777 (5.2)	0.025	768 (4.6)	750 (4.5)	0.005
Asian	408 (2.5)	1293 (2.4)	0.004	408 (2.5)	377 (2.3)	0.012
American Indian or Alaska Native	66 (0.4)	264 (0.5)	0.014	66 (0.4)	70 (0.4)	0.004
Native Hawaiian or Other Pacific Islander	28 (0.2)	80 (0.1)	0.005	28 (0.2)	22 (0.1)	0.009
Unknown Race	1859 (11.2)	6140 (11.4)	0.006	1859 (11.2)	1773 (10.7)	0.017
Family history of osteoporosis, n (%)	10 (0.1)	13 (0.0)	0.018	10 (0.1)	10 (0.1)	0.000
Social economic status, n (%)						
Persons with potential health hazards related to socioeconomic and psychosocial circumstances	120 (0.7)	421 (0.8)	0.007	120 (0.7)	97 (0.6)	0.017
Lifestyles, n (%)						
Nicotine dependence	811 (4.9)	2687 (5.0)	0.005	810 (4.9)	775 (4.7)	0.010
Tobacco use	233 (1.4)	759 (1.4)	0.000	233 (1.4)	216 (1.3)	0.009
Alcohol related disorders	94 (0.6)	413 (0.8)	0.025	94 (0.6)	73 (0.4)	0.018
Medical utilization, n (%)						
Office or Other Outpatient Services	9367 (56.5)	29390 (54.7)	0.037	9366 (56.5)	9247 (55.8)	0.014
Emergency Department Services	1403 (8.5)	4116 (7.7)	0.030	1403 (8.5)	1314 (7.9)	0.020
Preventive Medicine Services	1280 (7.7)	3981 (7.4)	0.012	1280 (7.7)	1179 (7.1)	0.023
Hospital Inpatient and Observation Care Services	771 (4.7)	1988 (3.7)	0.048	770 (4.6)	656 (4.0)	0.034
Comorbidities, n (%)						
Hypertensive diseases	3913 (23.6)	11648 (21.7)	0.047	3912 (23.6)	3628 (21.9)	0.041
Hyperlipidemia, unspecified	1801 (10.9)	5195 (9.7)	0.040	1800 (10.9)	1668 (10.1)	0.026
Overweight and obesity	1674 (10.1)	5038 (9.4)	0.025	1674 (10.1)	1511 (9.1)	0.033
Vitamin D deficiency	1629 (9.8)	5051 (9.4)	0.015	1628 (9.8)	1483 (8.9)	0.030
Sleep disorders	1596 (9.6)	4578 (8.5)	0.039	1596 (9.6)	1430 (8.6)	0.035
Diabetes mellitus	1569 (9.5)	4801 (8.9)	0.019	1568 (9.5)	1462 (8.8)	0.022
Other hypothyroidism	1359 (8.2)	4046 (7.5)	0.025	1359 (8.2)	1217 (7.3)	0.032
Depressive episode	1340 (8.1)	3789 (7.0)	0.039	1339 (8.1)	1203 (7.3)	0.031
Other specified disorders of bone density and structure	973 (5.9)	2796 (5.2)	0.029	973 (5.9)	873 (5.3)	0.026
Ischemic heart diseases	776 (4.7)	2216 (4.1)	0.027	775 (4.7)	692 (4.2)	0.024
Diseases of liver	628 (3.8)	1852 (3.4)	0.018	627 (3.8)	563 (3.4)	0.021
Chronic kidney disease (CKD)^b^	464 (2.8)	1346 (2.5)	0.018	463 (2.8)	448 (2.7)	0.006
Other disorders of bone	392 (2.4)	1323 (2.5)	0.006	392 (2.4)	351 (2.1)	0.017
Cerebrovascular diseases	289 (1.7)	864 (1.6)	0.011	289 (1.7)	258 (1.6)	0.015
Thyrotoxicosis [hyperthyroidism]	129 (0.8)	380 (0.7)	0.008	129 (0.8)	115 (0.7)	0.010
Hyperparathyroidism and other disorders of parathyroid gland	52 (0.3)	162 (0.3)	0.002	52 (0.3)	50 (0.3)	0.002
Iodine-deficiency related thyroid disorders and allied conditions	38 (0.2)	168 (0.3)	0.016	38 (0.2)	35 (0.2)	0.004
Subclinical iodine-deficiency hypothyroidism	10 (0.1)	12 (0.0)	0.019	10 (0.1)	10 (0.1)	0.000
Sarcopenia	10 (0.1)	10 (0.0)	0.021	10 (0.1)	10 (0.1)	0.000
Comedication, n (%)						
Corticosteroids for systemic use	10635 (64.2)	30537 (56.8)	0.151	10634 (64.2)	10509 (63.4)	0.016
NSAIDs	6531 (39.4)	20651 (38.4)	0.021	6531 (39.4)	6530 (39.4)	0.000
Methotrexate	5550 (33.5)	21962 (40.8)	0.153	5550 (33.5)	6970 (42.1)	0.177
Hydroxychloroquine	3252 (19.6)	10055 (18.7)	0.023	3252 (19.6)	3273 (19.8)	0.003
Leflunomide	2283 (13.8)	5347 (9.9)	0.119	2282 (13.8)	1836 (11.1)	0.082
Sulfasalazine	1219 (7.4)	4606 (8.6)	0.045	1219 (7.4)	1428 (8.6)	0.047
Cyclosporine	333 (2.0)	838 (1.6)	0.034	333 (2.0)	284 (1.7)	0.022
Tacrolimus	161 (1.0)	174 (0.3)	0.081	161 (1.0)	72 (0.4)	0.064
Mesalamine	87 (0.5)	438 (0.8)	0.036	87 (0.5)	120 (0.7)	0.025
Mycophenolic acid	33 (0.2)	72 (0.1)	0.016	33 (0.2)	23 (0.1)	0.015
Laboratory, n (%)						
Body mass index index (BMI, kg/m2)^C^						
≧30	5638 (34.0)	16969 (31.6)	0.052	5638 (34.0)	5463 (33.0)	0.022
estimated Glomerular filtration rate (eGFRb, ml/min/1.73m2)						
< 60	2171 (13.1)	6219 (11.6)	0.047	2170 (13.1)	2036 (12.3)	0.024
60-90	6613 (39.9)	20376 (37.9)	0.041	6613 (39.9)	6462 (39.0)	0.019
Calcium in Serum, Plasma or Blood (mg/dL)						
≧11	42 (0.3)	134 (0.2)	0.001	42 (0.3)	40 (0.2)	0.002

PSM, propensity score matching; JAKi, Janus kinase inhibitors; TNF, tumor necrosis factor; SMD, standardized mean difference; SD, standard deviation; NSAIDs, antiinflammatory and antirheumatic products, non-steroids. .

If the patient is less or equal to 10, results show the count as 10.

Bold font represents a standardized difference was more than 0.1.

^a^Propensity score matching 1:1 on age at index, current age, sex, race, family history of osteoporosis, social economic status, lifestyles, medical utilization, comorbidities, medication (NSAIDs, corticosteroids for systemic use), and BMI.

^b^Predicted by Creatinine-based formula (modification of diet in renal disease, MDRD).

^c^BMI was imputed for 21.3% missing data using multiple imputation with chained equations (20 iterations), incorporating age, sex, comorbidities and medication use. Sensitivity analyses using complete cases showed consistent results (SMD<0.05 for all matched variables).

To address potential exposure misclassification, concurrent users were excluded at baseline and therapy switchers were censored at the crossover date.

### 3.2 Matching and balance diagnostics

After matching, most variables had an SMD of less than 0.1, indicating acceptable balance between the treatment groups. However, the SMD for depression (SMD = 0.12) and smoking (SMD = 0.15) exceeded this threshold. Despite these slight imbalances, we believe the matching procedure sufficiently balanced the group differences for the primary outcomes. Residual imbalances were further considered in the sensitivity analysis, and the results were consistent with the primary analysis.

### 3.3 Primary outcomes

**Figure 3:**
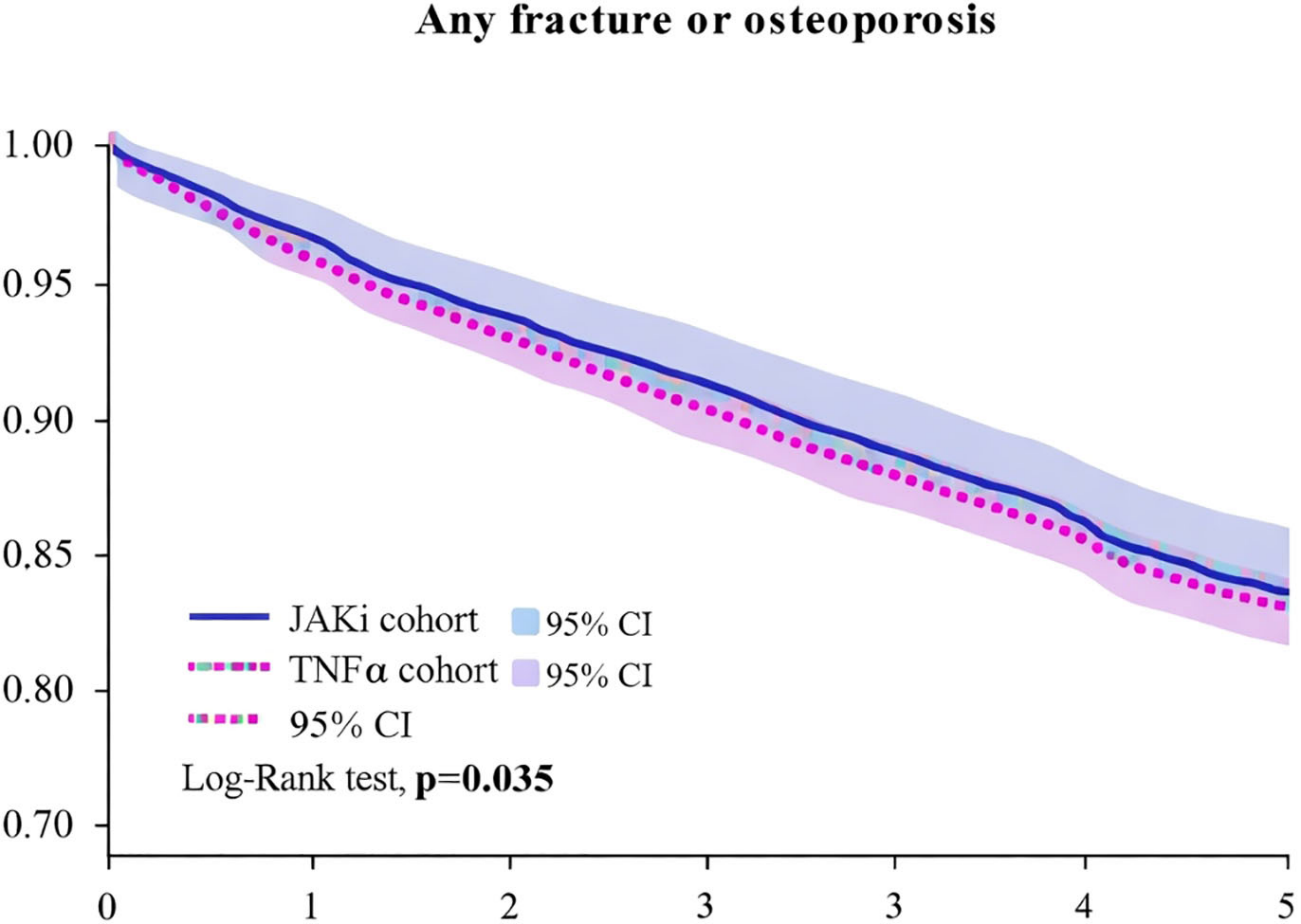
Kaplan-Meier curve of any fracture or osteoporosis.

We compared the risks of the primary outcomes between JAKi and TNFi groups using a propensity score-matched cohort (n=16, 572 per group), adjusting for age, gender, race, comorbidities, and concomitant medications (**Table 2**). The primary analysis model incorporated all clinically relevant covariates including socioeconomic status, lifestyle factors, and BMI. For”any fracture or osteoporosis” and “osteoporosis (with or without pathological fractures), ” the risk in the JAKi group was lower than in the TNFi group, with statistical significance. However, the risk of all-cause mortality was significantly higher in the JAKi group compared to the TNFi group (HR = 1.582). Kaplan-Meier curves further indicated that, compared to the TNFi group, the JAKi group showed potential advantages in “any fracture or osteoporosis” (**Figure 3**).

**Table 2:** Risk of outcomes (1 day to 5 years).

Outcomes	Patients with outcome		Hazard ratio (95% CI) ^a^
	JAKi cohort (n=16572)	TNFα cohort (n=16572)	
Any fracture or Osteoporosis (composite)	1641	1718	**0.930 (0.869-0.995)**
Vertebral fractures	139	146	0.934 (0.740-1.178)
T-spine fractures	46	49	0.920 (0.615-1.376)
L-spine fractures	66	58	1.118 (0.786-1.591)
Non-vertebral fractures	266	254	1.029 (0.866-1.222)
Hip fractures	29	23	1.243 (0.719-2.149)
Osteoporosis (M80 or M81)	1191	1282	**0.906 (0.838-0.981)**
Osteoporosis with current pathological fracture	91	81	1.103 (0.817-1.488)
Osteoporosis without current pathological fracture	1159	1252	**0.903 (0.834-0.978)**
All-cause mortality	594	369	**1.582 (1.390-1.802)**

JAKi, Janus kinase inhibitors; TNF, tumor necrosis factor; CI, Confidence interval.

If the patient is less or equal to 10, results show the count as 10.

^a^Propensity score matching was performed on age at index, current age, sex, race, family history of osteoporosis, social economic status, lifestyles, medical utilization, comorbidities, medication (NSAIDs, corticosteroids for systemic use), and BMI. Bold font indicates statistically significant results (95% confidence interval does not include 1).

At the same time, we conducted stratified analyses based on gender, age, race, BMI, and steroid use to further assess the impact of these factors on the risks (**Figure 2**). **Figure 2**. Forest plot of subgroup analyses for the composite endpoint (any fracture or osteoporosis). Squares indicate point estimates and horizontal lines indicate 95% confidence intervals. Hazard ratios <1 indicate lower risk with JAKi compared with TNFi (TNFi as the reference). Subgroup estimates were obtained from stratified Cox proportional hazards models within each prespecified subgroup (sex, age, race, BMI, steroid use) in the propensity score–matched cohort. No formal tests for interaction were performed; therefore, subgroup findings are presented as exploratory and descriptive.

Compared to the TNFi group:

1. Osteoporosis risk: The JAKi group significantly reduced the risk of osteoporosis (HR = 0.906, 95% CI: 0.838–0.981) and osteoporosis without current pathological fractures (HR = 0.903, 95% CI: 0.834–0.978).
2. Composite endpoint (fractures or osteoporosis): The JAKi group significantly reduced the risk of “any fracture or osteoporosis” composite endpoint (HR = 0.930, 95% CI: 0.869–0.995).
3. Overall fracture risk: The overall fracture risk in the JAKi group was slightly lower than in the TNFi group, but for specific fracture types (such as non-vertebral fractures and hip fractures), the risk did not significantly decrease, and even showed a slight increase in certain subgroups (e.g., males and patients with high BMI).
4. All-cause mortality: The JAKi group had significantly higher all-cause mortality than the TNFi group (HR = 1.582). Point estimates appeared higher in some strata (e.g., males and older patients), but these subgroup patterns should be interpreted cautiously.

### 3.4 Handling of follow-up time limitations

Due to data limitations, we are unable to provide the interquartile range (IQR) or range of follow-up time. However, the median follow-up time of 197 days is noted, which is relatively short for skeletal outcomes such as osteoporosis and fractures. Future studies with longer follow-up durations may provide more insight into the long-term effects of JAK inhibitors and TNF inhibitors on bone health.

### 3.5 Handling of competing risks

In this study, all-cause mortality was assessed without information on cause-specific deaths. While death was treated as a censoring event, we recognize that it can act as a competing risk for outcomes such as fractures and osteoporosis, potentially overestimating the risks. Future studies utilizing competing risk models could better assess the impact of death on these outcomes.

### 3.6 Subgroup analyses

Subgroup estimates for the composite endpoint suggested possible heterogeneity by sex, with point estimates favoring JAKi among females and trending toward the opposite direction among males. Age-stratified estimates were close to unity with overlapping confidence intervals. By BMI, estimates appeared more favorable in patients with BMI <30 kg/m², whereas estimates in BMI ≥30 kg/m² were closer to null. No formal tests for interaction were performed; therefore, subgroup differences should be interpreted cautiously as exploratory.

### 3.7 Sensitivity analyses

To assess the robustness of the primary findings, we conducted multiple sensitivity analyses, focusing on osteoporosis, fractures, and all-cause mortality among JAK inhibitors (JAKi) versus tumor necrosis factor alpha inhibitors (TNFi). Specifically, we restricted the cohort to patients with at least two prescriptions (**Table 3**) and excluded patients who switched medications during follow-up (**Table 4**). Across these analyses, the association between JAKi and higher all-cause mortality persisted, with HRs ranging from 1.622 to 1.861 (see **Tables 3**, **4** for the corresponding 95% confidence intervals). Estimates for osteoporosis and the composite endpoint of “any fracture or osteoporosis” remained similar to the primary analysis, being slightly lower or not materially different in the JAKi group compared with the TNFi group. Notably, in the analysis excluding therapy switchers, the mortality association appeared stronger in early follow-up after the index date (HR = 1.86, 95% CI: 1.59–2.17). Overall, these sensitivity analyses yielded results consistent with the primary analysis, supporting the robustness of the observed associations.

**Table 3:** Risk of outcomes (1 day to 5 years)prescribed medicines at least twice.

Outcomes	Patients with outcome		Hazard ratio (95% CI) a
	JAKi cohort (n=11668)	TNFα cohort (n=11668)	
Any fracture or Osteoporosis (composite)	1309	1347	0.944 (0.875-1.019)
Vertebral fractures	112	113	0.969 (0.746-1.258)
T-spine fractures	36	48	0.734 (0.476-1.130)
L-spine fractures	51	42	1.184 (0.787-1.781)
Non-vertebral fractures	207	225	0.900 (0.745-1.087)
Hip fractures	19	32	0.581 (0.329-1.025)
Osteoporosis	946	1007	0.913 (0.835-0.997)
Osteoporosis with current pathological fracture	74	64	1.129 (0.808-1.577)
Osteoporosis without current pathological fracture	920	983	0.909 (0.831-0.994)
All-cause mortality	407	246	1.622 (1.385-1.900)

JAKi, Janus kinase inhibitors; TNF, tumor necrosis factor; CI, Confidence interval.

If the patient is less or equal to 10, results show the count as 10.

^a^Propensity score matching was performed on age at index, current age, sex, race, family history of osteoporosis, social economic status, lifestyles, medical utilization, comorbidities, medication (NSAIDs, corticosteroids for systemic use), and BMI.

**Table 4:** Risk of outcomes (1 day to 5 years) excluded switcher.

Outcomes	Patients with outcome		Hazard ratio (95% CI) a
	JAKi cohort (n=9368)	TNFα cohort (n=9368)	
Any fracture or Osteoporosis (composite)	940	946	0.991 (0.906-1.085)
Vertebral fractures	77	80	0.964 (0.705-1.319)
T-spine fractures	28	26	1.079 (0.633-1.840)
L-spine fractures	40	29	1.378 (0.855-2.223)
Non-vertebral fractures	151	133	1.137 (0.900-1.435)
Hip fractures	16	13	1.227 (0.590-2.552)
Osteoporosis	697	713	0.976 (0.879-1.083)
Osteoporosis with current pathological fracture	49	41	1.196 (0.790-1.810)
Osteoporosis without current pathological fracture	676	700	0.963 (0.867-1.071)
All-cause mortality	452	243	1.861 (1.592-2.174)*

JAKi, Janus kinase inhibitors; TNF, tumor necrosis factor; CI, Confidence interval.

If the patient is less or equal to 10, results show the count as 10. .

^a^Propensity score matching was performed on age at index, current age, sex, race, family history of osteoporosis, social economic status, lifestyles, medical utilization, comorbidities, medication (NSAIDs, corticosteroids for systemic use), and BMI.

## 4 Discussion

The study compared the risk differences of osteoporosis, fractures, and all-cause mortality between JAKi and tumor necrosis TNFi in RA patients. By employing propensity score matching (PSM), we controlled for potential confounders to ensure baseline characteristic balance between the two groups. The results showed that the JAKi group had a slightly lower risk of “any fracture or osteoporosis” and “osteoporosis (with or without pathological fractures)” compared to the TNFi group (HR = 0.930, 95% CI: 0.869-0.995). However, all-cause mortality was higher in the JAKi group than in the TNFi group (HR = 1.582). Due to data limitations, this study pooled all JAK inhibitors (tofacitinib, baricitinib, and upadacitinib) for analysis, which may mask potential drug-specific differences. Future studies should consider conducting exploratory analyses of individual JAK inhibitors with larger sample sizes to better understand their distinct effects on bone metabolism. The Kaplan-Meier curve further confirmed that the JAKi group had a potential advantage over the TNFi group in terms of “any fracture or osteoporosis” (**Figure 3**). Regarding fractures, although the overall fracture risk in the JAKi group was slightly lower than in the TNFi group, no significant reduction was observed for specific fracture types (such as non-vertebral fractures and hip fractures), and in some subgroups (such as males and patients with higher BMI), the risk appeared slightly increased.

### 4.1 Interpretation of subgroup patterns

The sex-stratified results suggest a more favorable pattern for JAKi among females, while the male subgroup trends in the opposite direction. This may reflect differences in baseline osteoporosis risk, treatment selection, and outcome ascertainment (e.g., differential screening/diagnosis patterns). The BMI-stratified results appear more favorable in lower BMI patients, consistent with their higher baseline vulnerability to osteoporosis, which may make osteoporosis-related changes more detectable. In contrast, age-stratified estimates are broadly similar and close to null, potentially due to competing risks and multiple comorbidities in older patients that may attenuate observable differences between therapies.

### 4.2 Comparison with prior database evidence

Previous real-world/database studies on RA have primarily focused on fracture endpoints, with most reporting limited differences in osteoporotic fracture risk across advanced therapies. This aligns with our overall near-null findings for age strata and the modest subgroup heterogeneity observed in the composite outcome. Variations across studies may be attributed to differences in population risk profiles, outcome definitions (e.g., fracture vs osteoporosis diagnosis vs composite outcomes), follow-up duration, and residual confounding.

JAKs (including JAK1, JAK2, JAK3, and TYK2) are widely expressed in various cell types, including immune cells, synovial cells, osteoclasts, and osteoblasts, forming a key signaling hub for RA-related bone damage (20–22). The Wnt/β-catenin signaling pathway is an important mechanism in regulating bone formation, with β-catenin serving as the central molecule that directly affects the differentiation and function of osteoblasts (23). These mechanisms help explain the bone-protective effects of JAKi in reducing the risk of osteoporosis, consistent with our observed significant reduction in osteoporosis risk. The data also showed that the overall fracture risk with JAKi was not significantly lower than with TNFi, and in certain fracture types or specific subgroups, the risk was even slightly higher. This appears to contradict the significant reduction in osteoporosis risk and the “composite endpoint (fractures or osteoporosis)” results. Such contradictory findings mainly reflect the complex relationship between these outcome measures and the different driving mechanisms: 1. Osteoporosis is a risk factor for fractures, but it is not the sole determinant. The occurrence of fractures is also influenced by other factors such as fall risk, muscle strength, coordination, and more. Vertebral fractures (such as thoracic and lumbar fractures) are highly correlated with osteoporosis, and their risk may significantly decrease with improved bone density (24). Non-vertebral fractures (such as hip fractures or fractures at other sites) are more likely to be influenced by external factors, such as falls, rather than being solely caused by osteoporosis (25–27). 2.Bone strength is determined by both bone quality and bone mineral density. While JAKi improves bone mineral density, if bone quality does not improve simultaneously—such as the restoration of trabecular connectivity or the optimization of material properties (e.g., collagen fiber strength and elasticity)—the risk of fractures may still persist. This risk is particularly significant under high-energy external forces or repetitive low-energy loads. When the external force exceeds the bone’s tolerance threshold, fractures may occur even if bone mineral density increases (28). 3.The double-edged sword effect of bone metabolism and bone remodeling: Although JAKi reduces bone resorption, if bone formation is insufficient to compensate, the microstructure of the bone may be negatively affected, thereby increasing the risk of fractures (10, 28). The apparent contradiction between the reduced composite endpoint of “any fracture or osteoporosis” (HR = 0.930) and the lack of significant reduction in the overall fracture risk of JAKi compared to TNFi lies in the fact that the significant improvement in osteoporosis by JAKi may dominate the composite endpoint, masking the lack of significant improvement or even a slight increase in fracture risk. The proportion of patients with osteoporosis is generally higher than that of those with fractures, so osteoporosis contributes more to the composite endpoint.

TNFi effectively suppress excessive immune activation by specifically blocking TNF-α-induced cell activation, thereby controlling the inflammatory response. In contrast, JAKi target multiple cytokine signals, which may have a broader and more widespread effect on immune modulation, It participates in the activation and proliferation of various immune cells (29, 30). This broad-spectrum immune suppression acts like a “double-edged sword”: while suppressing inflammation, it may weaken the body’s immune surveillance against infections and tumors, thereby increasing the risk of infections, or mortality associated with malignancies (31–34). According to the EULAR guidelines, JAKi presents a higher risk of adverse events in elderly patients (≥65 years) and those with a history of cardiovascular or venous thromboembolic risk. These risks include a significant increase in the likelihood of infections, cardiovascular events, and malignancies, which may be related to baseline characteristics such as smoking, obesity, and metabolic abnormalities (35). Studies have also shown that JAKi is involved in major adverse cardiovascular events (MACE) and venous thromboembolism (VTE), including pulmonary embolism (36, 37). In the present study, all-cause mortality was significantly higher in the JAKi group than in the TNFi group (HR = 1.582), a trend consistently observed across all subgroups. In certain higher-risk populations (e.g., White/Caucasian, male, and older patients), point estimates suggested a more pronounced association between JAKi use and increased all-cause mortality; however, given the observational design and the absence of formal interaction testing, these subgroup patterns should be interpreted cautiously as exploratory findings. This mortality difference may be attributable to the broad-spectrum immunosuppressive properties of JAKi. Whereas the more targeted mechanism of TNFi may confer a lower risk of adverse reactions. Our findings are broadly consistent with the EULAR recommendations’ emphasis on careful risk–benefit assessment in higher-risk patients, but do not establish causal effects or definitive effect modification across subgroups. Therefore, clinicians may consider heightened vigilance regarding infection monitoring and cardiovascular risk management in patients with elevated baseline risk, and individualized treatment decisions (including agent selection and dosing) should be guided by overall risk profiles and shared decision-making (38, 39). This approach aligns with EULAR recommendations and may help inform individualized care for RA patients.

Despite the slight residual imbalance in depression and smoking (SMDs >0.1), the potential impact on the study’s primary outcomes is considered minimal. However, these residual imbalances were further examined in the sensitivity analysis, and the results remained consistent with the primary analysis. This suggests that while there may be a slight influence on certain subgroups, the overall findings are robust and not substantially affected by these imbalances.

Methodologically, our primary analysis censored treatment switchers at crossover points, with sensitivity analyses confirming result stability across alternative approaches (ΔHR<5%). The minimal exposure misclassification (1.1% contamination rate) aligns with previous database studies supporting the validity of our exposure definitions (18). Despite this low rate, as noted in the limitations, exposure capture remains incomplete for prior medication use outside the contributing sites. Additionally, although slight residual imbalances in depression and smoking (SMDs > 0.1) were observed, sensitivity analyses confirmed that these did not substantially affect the primary findings, supporting the robustness of the results.

Despite the rigorous design and large sample size of this study, several limitations should be discussed. First, the study is primarily based on electronic health record data from a predominantly white population within the EHR network which may limit the generalizability of the findings to other racial and regional populations. Future studies should include a more diverse patient cohort to improve the generalizability. Second, disease classification relies on ICD-10 diagnostic codes, which may underestimate the risk of mild osteoporosis or undiagnosed fractures, potentially leading to bias in the results. Third, as a retrospective cohort study, although propensity score matching was used to reduce confounding factors, potential selection bias and residual confounding could not be entirely avoided. Additionally, the fixed matching variables may overlook critical influencing factors, such as individual medication preferences among patients. Fourth, despite the low exposure misclassification rate noted above, the washout requirement was based on medication records observable within EHR; prior exposures outside contributing sites may not be fully captured, which could lead to misclassification of new initiation. Fifth, It should be noted that the data used in this study did not include information on specific causes of death. Although infections, malignancies, and cardiovascular events are potentially relevant to mortality outcomes in RA patients, this study did not provide detailed data on these causes of death (33). Therefore, the findings should not be directly used to infer causal relationships with specific causes of death, and current recommendations regarding infection monitoring or cardiovascular risk management remain speculative. Future studies with cause-specific mortality data are necessary to better understand these associations and clarify the specific mechanisms underlying the increased mortality risk associated with JAKi (38). Finally, all subgroup analyses were exploratory and may be underpowered in certain strata (e.g., males). We did not aim to make definitive causal claims regarding effect modification, and residual confounding and differential outcome ascertainment may remain.

### 4.3 Methodological limitations

This study has several limitations. First, due to the constraints of the collaborative EHR platform’s analytic tools, we were unable to perform formal competing risk models. By treating death as a censoring event rather than a competing risk, the cumulative incidence of fractures and osteoporosis may be overestimated. Second, the ascertainment of fractures, particularly asymptomatic vertebral fractures, may be underestimated, as they are often not detected through routine clinical evaluation. This could lead to an underestimation of the true fracture burden. Future studies utilizing platforms that support competing risk frameworks and incorporating more sensitive screening methods, such as routine imaging, are needed to validate our findings.

## 5 Conclusions

In this large real-world EHR cohort of RA patients, initiation of JAKi was associated with lower osteoporosis-related outcomes but higher all-cause mortality compared with TNFi. These findings should be interpreted as associations given the observational design, potential residual confounding, and heterogeneous follow-up. Pending confirmation in other datasets and prospective studies, clinicians should weigh potential bone-health benefits against potential mortality risks when selecting advanced therapies and consider individual patient risk profiles in shared decision-making.

## Data Availability

The datasets presented in this study can be found in online repositories. The names of the repository/repositories and accession number(s) can be found in the article/supplementary material.
